# Manual therapies for primary chronic headaches: a systematic review of randomized controlled trials

**DOI:** 10.1186/1129-2377-15-67

**Published:** 2014-10-02

**Authors:** Aleksander Chaibi, Michael Bjørn Russell

**Affiliations:** 1 Head and Neck Research Group, Research Centre, Akershus University Hospital, 1478 Lørenskog, Oslo, Norway; 2 Institute of Clinical Medicine, Akershus University Hospital, University of Oslo, 1474 Nordbyhagen, Oslo, Norway

**Keywords:** Randomized clinical trials, Primary chronic headache, Manual therapies, Massage, Physiotherapy, Chiropractic

## Abstract

This is to our knowledge the first systematic review regarding the efficacy of manual therapy randomized clinical trials (RCT) for primary chronic headaches. A comprehensive English literature search on CINHAL, Cochrane, Medline, Ovid and PubMed identified 6 RCTs all investigating chronic tension-type headache (CTTH). One study applied massage therapy and five studies applied physiotherapy. Four studies were considered to be of good methodological quality by the PEDro scale. All studies were pragmatic or used no treatment as a control group, and only two studies avoided co-intervention, which may lead to possible bias and makes interpretation of the results more difficult. The RCTs suggest that massage and physiotherapy are effective treatment options in the management of CTTH. One of the RCTs showed that physiotherapy reduced headache frequency and intensity statistical significant better than usual care by the general practitioner. The efficacy of physiotherapy at post-treatment and at 6 months follow-up equals the efficacy of tricyclic antidepressants. Effect size of physiotherapy was up to 0.62. Future manual therapy RCTs are requested addressing the efficacy in chronic migraine with and without medication overuse. Future RCTs on headache should adhere to the International Headache Society’s guidelines for clinical trials, i.e. frequency as primary end-point, while duration and intensity should be secondary end-point, avoid co-intervention, includes sufficient sample size and follow-up period for at least 6 months.

## Introduction

Primary chronic headaches i.e. chronic migraine (CM), chronic tension-type headache (CTTH) and chronic cluster headache has significant health, economic and social costs. About 3% of the general population suffers from chronic headache with female predominance [1]. The International Classification of Headache Disorders III β (ICDH-III β) defines CM as ≥15 headache days/month for at least 3 months with features of migraine in ≥8 days/month, CTTH is defined as on average ≥15 days/month with tension-type headache for at least 3 months, and chronic cluster headache as attacks at least every other day for more than 1 year without remission, or with remissions lasting <1 month [2].

About 80% consult their primary physician for primary chronic headache [3], and pharmacological management is considered first line of treatment. However, the risk is that it may cause overuse of acute headache medication due to frequent headache attacks. 47% of those with primary chronic headache in the general Norwegian population overused acute headache medication [1,4]. Considering the high use of acute medication, both prophylactic medication and non-pharmacological management should therefore be considered in the management [5,6]. Prophylactic medication is used only by 3% in the general Norwegian population, while 52% have tried physiotherapy and 28% have tried chiropractic spinal manipulative therapy [3]. Non-pharmacological management has furthermore the advantage of few and usually minor transient adverse events and no pharmacological interaction/adverse event [7].

Previous systematic reviews have focused on RCTs for tension-type headache, migraine and/or cervicogenic headache, but not on efficacy on primary chronic headache [5,6,8-11]. Manual therapy is a physical treatment used by physiotherapists, chiropractors, osteopaths and other practitioners to treat musculoskeletal pain and disability, and includes massage therapy, joint mobilization and manipulation [12].

This is to our knowledge the first systematic review assessing the efficacy of manual therapy randomized controlled trials (RCT) for primary chronic headache using headache frequency as primary end-point and headache duration and intensity as secondary end-points.

## Review

### Methods

The English literature search was done on CINHAL, Cochrane, Medline, Ovid and PubMed. Search words were; migraine, chronic migraine, tension-type headache, chronic tension-type headache, cluster headache, chronic cluster headache combined with the words; massage therapy, physiotherapy, spinal mobilization, manipulative therapy, spinal manipulative therapy, osteopathic treatment or chiropractic. We identified studies by a comprehensive computerized search. Relevant reviews were screened for additional relevant RCTs. The selection of articles was performed by the authors. All RCTs written in English using either of the manual therapies for CM, CTTH and/or chronic cluster headache were evaluated. Studies including combined headache types without specific results for CM, CTTH and/or chronic cluster headache were excluded. The review included manual therapy RCTs presenting at least one of the following efficacy parameters; headache frequency, duration and pain intensity for CM, CTTH and/or chronic cluster headache as recommended by the International Headache Society’s clinical trial guidelines [13,14]. Headache frequency is a primary end-point, while duration and pain intensity are secondary end-points. Headache diagnoses were preferentially classified according to the criteria of ICHD-III β or previous editions [2,15-17]. The methodological quality of the included RCTs was evaluated using the PEDro scale, Table 1[18]. A RCT was considered to be of high quality if the PEDro score was ≥6 of a maximum score of 10. The methodological quality of the RCTs was assessed by AC. The PRISMA 2009 checklist was applied for this systematic review. Effect size was calculated when possible. Effect size of 0.2 was regarded as small, 0.5 as medium and 0.8 as large [19].

**Table 1:** PEDro score “yes” or “no” items

1.	Subjects were randomly allocated to groups.
2.	Allocation was concealed.
3.	The groups were similar at baseline regarding the most important prognostic indicators.
4.	There was blinding of all subjects.
5.	There was blinding of all therapists who administered the therapy.
6.	There was blinding of all assessors who measured at least one key outcome.
7.	Measurements of at least one key outcome were obtained from more than 85% of the subjects initially allocated to groups.
8.	All subjects for whom outcome measurements were available received the treatment or control condition as allocated, or where this was not the case, data for at least one key outcome were analyzed by “intention to treat”.
9.	The results of between-groups statistical comparisons are reported for at least one key outcome.
10.	The study provides both point measurements and measurements of variability for at least one key outcome measure.

The maximum score is 10 if all 10 items can be replied yes.

This systematic review was executed directly based on the ascertained RCTs available and has not been registered as a review protocol.

### Results

The literature search identified six RCTs that met our inclusion criteria. One study applied massage therapy (MT) and five studies applied physiotherapy (PT) [20-25]. All studies assessed CTTH, while no studies assessed CM or chronic cluster headache.

#### Methodological quality

Table 2 shows that the methodological PEDro score of the included RCTs ranged from 1 to 8 points. Four RCTs were considered of good methodological quality, while two RCTs had lower scores.

**Table 2:** The methodological PEDro score of the included randomized controlled trials (RCTs)

	**1**	**2**	**3**	**4**	**5**	**6**	**7**	**8**	**9**	**10**	**PEDro score**
Toro-Velasco [20]	Yes	Yes	Yes	No	No	Yes	Yes	Yes	Yes	Yes	8/10
Jay [21]	No	No	No	No	No	No	No	No	Yes	No	1/10
Demirturk [22]	Yes	Yes	Yes	No	No	No	No	No	Yes	Yes	5/10
Torelli [23]	Yes	Yes	Yes	No	No	No	Yes	No	Yes	Yes	6/10
Ettekoven [24]	Yes	Yes	Yes	No	No	Yes	Yes	Yes	Yes	Yes	8/10
Castien [25]	Yes	Yes	Yes	No	No	No	Yes	Yes	Yes	Yes	7/10

#### Randomized controlled trials (RCT)

Table 3 shows the study population, intervention and efficacy of the six RCTs.

**Table 3:** Results of manual therapy randomized controlled trials (RCTs) of chronic tension-type headache (CTTH)

**Country**	**Study population**	**Method**	**Intervention**	**Results**
**Spain** 2009 [20]	11 participants (3 M, 8 F)	RCT of 8 days duration conducted by a physiotherapist i.e.	All interventions received two treatments over 8 days lasting for 40 min	Headache intensity decreased statistical significant 24 hours after massage (p < 0.05), whereas detuned ultrasound had no statistical significant effect
	Age 41-60 yrs	2 treatments with one week interval, each followed by 24-hours post-treatment assessment	Head and neck massage	Mean headache intensity was reduced 24% after massage, and 3% after detuned ultrasound
	Mean 51 ± 15 yrs	Comparison of pre-and post-treatment at the treatment laboratory	Detuned ultrasound at head and neck area (control group)	Effect size 0.32
	Headache diagnosed by neurologist		Drop outs (n = 0)	
**USA** 1989 [21]	55 participants (15 M, 40 F)	RCT of 9 months duration conducted by a physiotherapist i.e. 3 months treatment 3, 6 months follow-up	All interventions received detoxification (when necessary) and amitriptyline (in some cases)	The efficacy parameter was the mean headache index defined as weekly frequency x intensity on a 0-10 numeric rating. Mean headache index at baseline was 30
	Age 23-62 yrs	Comparison of weekly treatments and follow-up	Biofeedback treatment (n = 9; 4 M and 5 F)	Biofeedback reduced the headache index defined as frequency x intensity by 83% and 76% post-treatment and 3 months follow-up, while physiotherapy combined with TENS reduced it by 97% and 93% and TENS by 94% and 92% respectively
	Mean age 36.9 yrs	Headache diary recordings	Physiotherapy including heat pack, soft tissue work and ultrasound with home exercises and home transcutaneus electrical nerve stimulation (TENS) treatment, average 12.8 sessions over 3 months (n = 28; 4 M and 24 F)	The improvement was sustained at 6 months in physiotherapy combined with TENS and TENS group, i.e. 95% and 97% reduction of the headache index
	Mean headache duration 8.4 yrs		TENS treatment by physiotherapist, 12 sessions followed by additional home TENS treatment twice a day for another month (n = 18; 7 M and 11 F)	
	Retrospect headache diagnosis		Drop outs of 6 months follow-up (n = 18; 7 M and 11 F)	Effect size not applicable
	12 participants had co-occurrence of menstrual migraine			
**Turkey** 2002 [22]	35 female participants	RCT of 10 weeks duration conducted by a physiotherapist i.e. 2 weeks baseline, 4 weeks treatment, 4 weeks follow-up	All interventions received superficial heat and classic massage to the neck and upper back prior to intervention	Efficacy parameter was headache index defined as headache frequency x intensity
	Age 19-59 yrs	Comparison of baseline, post-treatment and follow-up	Spinal connective tissue manipulation by a physiotherapist daily (n = 15)	Both the spinal connective tissue manipulation and cervical mobilization groups had statistical significant improvement, i.e. 38% vs. 54% post-treatment and 48% vs. 86% at 3 months follow-up
	Mean age 37.9 yrs	Headache diary recordings	Cervical mobilization according to Cyriax principle by a physiotherapist three times per week (n = 15)	No statistical significant differences between the two groups
	Mean headache duration 9.8 yrs		Drop outs (n = 5)	Effect size 0.21 before to after treatment, 0.30 after treatment to follow-up, and 0.37 before treatment to follow-up
	Headache diagnosed by neurologist			
**Denmark** 2004 [23]	CTTH (n = 24) (10 M, 14 F)	RCT of 6 months duration conducted by a physiotherapist i.e. 1 month baseline, 2 months treatment, 3 months follow-up	Standardised physiotherapy including massage, relaxation and stretching twice a week for 4 weeks followed by twice a week physical group exercise for 4 weeks (n = 18)	Headache days were statistical significantly reduced in the physiotherapy group post-treatment and at follow-up as compared to the observation group (p < 0.001)
	Age 24-63	Comparison of baseline, post-treatment and follow-up	Observation period, kept diary for 8 weeks followed by the same intervention (n = 19)	54% of participants responded with >50% reduction of headache days
	Mean age 44.9 yrs	Headache diary recordings	Drop outs (n = 11) (5 M, 6 F)	Headache duration and intensity were unchanged in both groups
	Duration of headache 23.1 yrs			Women responded statistical significantly better than men (p = 0.01)
	Mean headache frequency 15.8 days per month			Effect size not applicable
	Headache diagnosed by a neurologist			
**Netherland** 2006 [24]	TTH (n = 42) and episodic tension-type headache (n = 39)	RCT of 7½ months duration conducted by twenty physiotherapist’s i.e. Initial baseline 6 weeks treatment, 6 months follow-up, Comparison of pre- and post-treatment and follow-up	Craniocervical training program by a physiotherapist including low-load endurance exercises for cervicoscapular and craniocervical region with twice daily home exercise combined with postural correction exercises (n = 39)	Both groups had statically significant post-treatment improvement of headache frequency, duration and intensity
	Mean age 45.9 yrs	Headache diary recordings	Physiotherapy including massage, oscillation techniques described by Maitland and postural correction (n = 40)	In the craniocervical training group 82% and 85% had ≥50% reduction in headache frequency post-treatment and at follow-up, as compared to 52% and 35% in the physiotherapy group.
	Mean headache frequency 20.7 days per month		Drop outs (n = 2)	The effect was a statistical significant better in those with CTTH as compared to those with episodic tension-type headache (p < 0.0001).
	Headache diagnosed by physician			No individual data are presented for CTTH
				Effect size not applicable
**Netherland** 2011 [25]	82 participants (18 M, 64 F)	RCT of 8½ months duration conducted by four physiotherapist’s i.e. 2 weeks baseline, 2 months treatment, 6 months follow-up	Usual care by general practitioner which consisted of information, re-assurance, advise, lifestyle changes and prescription of analytics and NSAIDs if necessary 2-3 visits (n = 37)	The manual therapy group had statistical significant improvement of headache frequency, duration and intensity post-treatment and at follow-up as compared to usual care group (p < 0.001, p = 0.003, and p = 0.013). This effect was maintained at follow-up (p < 0.001 and p = 0.027), except for duration.
	Age 20-63 yrs	Comparison of baseline, post-treatment and follow-up	Manual therapy by a physiotherapist consisting of mobilization of cervical and thoracic column, exercises and postural correction for max 9 sessions of 30 min (n = 38)	At post-treatment and follow-up the headache frequency was reduced 77% and 77% in the manual therapy group as compared to 23% and 35% in usual care group.
	Mean age 40.4 yrs	Headache diary recordings	Drop outs (n = 7)	At post-treatment and at follow-up 88% and 82% of the manual therapy group had >50% reduction in headache frequency, and 28% and 41% of the usual care group.
	Mean headache duration 12.8 yrs			At post-treatment and follow-up duration was reduced 46% and 55% in the manual therapy group as compared to 5% and 27% in the usual care group.
	Mean headache frequency 24 days per month			At post-treatment and follow-up intensity was reduced 43% and 49% in the manual therapy group as compared to 16% and 30% in the usual care group.
	Headache diagnosed by physician			Frequency, duration and intensity effect size was 0.62, 0.27 and 0.44 at post-treatment and 0.50, 0.19 and 0.26 at follow-up
	21 participants had co-occurrence of migraine			

#### Massage therapy

A Spanish physiotherapist conducted a 2-armed prospective crossover RCT with pairwise comparisons and blinded outcome measures [20]. The study included participants with CTTH diagnosed by a neurologist. The ICHD-II criteria for CTTH were slightly modified, i.e. pain intensity was defined as ≤5 on a 0-10 numeric pain rating scale, and the accompanying symptoms photophobia, phonophobia or mild nausea was not allowed [16]. Primary and secondary end-points were not specified. Results are shown in Table 3.

#### Physiotherapy

An American 3-armed retrospectively RCT had unblinded outcome measures [21]. The diagnostic criteria were ≥25 headache days/month for >6 months without associated symptoms nausea, vomiting, photo- and phonophobia, but with tender muscles, i.e. CTTH with pericranial tenderness. Participants with cervicogenic headache or neurological findings were excluded. Primary and secondary end-points were not pre-specified, but headache index, defined here as headache frequency × severity, was the evaluated end-point.

A Turkish study conducted a 2-armed prospective RCT with unblinded outcome measures [22]. The participants were diagnosed with CTTH according to ICHD-I [15]. Participants with mixed headache, neurological and systemic aliment, or participants whom had received physiotherapy within 6 months prior to the study were excluded. Primary end-points was headache index defined as frequency × severity.

A Danish study conducted a 2-armed prospective RCT with blinded outcome measures [23]. Participants were diagnosed CTTH by a neurologist according to the criteria of ICHD-I [15]. Participants with other primary headaches, neuralgia, neurological, systemic or psychiatric disorders or medication overuse defined as >100 analgesic tablets or >2 doses of triptans and ergotamine per month were excluded. The primary end-point was headache frequency, and the secondary end-points were headache duration and intensity. The results shown in Table 3 were not influenced by pericranial muscles tenderness.

A Dutch study conducted a 2-armed prospective, multicentre RCT with blinded outcome measures [24]. Participants were diagnosed with CTTH by a physician according to ICHD-I [15]. Participants with multiple headache types or those whom had received physiotherapy within the last 6 months were excluded. Primary end-points were headache frequency while duration and intensity were secondary end-points.

The 2^nd^ Dutch study conducted a 2-armed prospective pragmatic, multicentre RCT with self-reported primary and secondary end-points, i.e. headache frequency, duration and intensity [25]. Participants were diagnosed by a physician according to the criteria of ICHD-II [16]. Participants with rheumatoid arthritis, suspected malignancy, pregnancy, non-Dutch speaking, those whom had received physiotherapy within the last 2 months, triptan, ergotamine or opiods users were excluded.

### Discussion

The current systematic review evaluating the efficacy of manual therapy in RCTs for primary chronic headaches only identified RCTs treating CTTH. Thus, the efficacy of CM and chronic cluster headache could not be evaluated in this review.

#### Methodological considerations

The methodological quality of studies assessing manual therapies for headache disorders are frequently being criticised for being too low. Occasionally rightly so, but often do the methodological design prevent manual therapy studies from reaching what is considered gold standard in pharmacological RCTs. For instance, a placebo treatment is difficult to establish while the investigator cannot be blinded for its applied intervention. The average score of the included studies was 5.8 (SD 2.6) points and four studies were considered of good quality. All RCTs failed to include sample size ≥50 in the smallest group. Sufficient sample size with power calculation prior is important to confine type 2 errors. Three studies did not state primary and secondary end-points, which confound effect-size calculation, and risk of type 2 errors inferred from multiple measures [20-22]. Conducting a manual therapy RCT is both time and cost consuming, while blinding often is difficult as there is no single validated standardized sham-treatment which can be used as a control group to this date. Thus, all of the included studies were pragmatic or used no treatment as a control group.

Apart from the participants in the retrospective study [21], all participants were diagnosed by a physician or neurologist. A diagnostic interview is the gold standard, while questionnaire and lay interviews are less precise diagnostic tools regarding headache disorders [26].

Co-intervention was only avoided in two studies [22,20]. Two studies performed intention-to-treat analysis which is recommended to protect against odd outcome values and preserve baseline comparability [24,25,27].

#### Results

The massage therapy study included only 11 participants, but the massage group had significantly more reduction in their headache intensity than detuned ultrasound group [20].

54%, 82% and 85% of the participants in three of the physiotherapy RCTs had a ≥50% reduction in headache frequency post-treatment [23-25], and the effect was maintained in the two studies that had a 6 months follow-up [24,25]. This is comparable with the 40-70% of participants whom have a similar effect using tricyclic antidepressants [28,29]. The effect of tricyclic also seems to improve over time, i.e. after more than 6 months treatment [29]. However, tricyclic antidepressants have a series of side effects in contrast to physiotherapy, while manual therapy requires more consultations. Two studies assessed headache index defined as headache frequency × intensity [21,22]. Both studies showed a significant improvement post-treatment and at 1 month and 6 months follow-up respectively.

Four of the studies reported 10.1 mean years with headache, thus, the effect observed is likely to be due to the therapeutic effect rather than spontaneous improvement or regression to the mean [21-23,25].

Acute headache medication is frequently used for primary headaches, and if the headache frequency increases, there is an increased risk for medication overuse headache. Increased use of prophylactic medication has thus been suggested in the management for primary chronic headaches [3]. Since manual therapies seems to have a beneficial effect that equals the effect of prophylactic medication [28,29], without the pharmacological side effects, manual therapies should be considered on an equal level as pharmacological management strategies.

Effect size could be calculated in three of the six RCTs. Effect size on headache frequency was up to 0.62, while it was less regarding duration and intensity, while headache index (frequency × intensity) was up to 0.37 (Table 3). Thus, a small to moderate effect size might however, be substantial to the individual, especially considering that nearly daily headache i.e. mean 12/14 days reduced to mean 3/14 days [25], which equals ≥75% reduction in headache frequency. Usually a ≥50% reduction is traditionally used in pain trails, but considering the fact that CTTH is difficult to treat, some investigators operate with ≥30% improvement of primary efficacy parameter compared with placebo [30].

#### Limitations

The present study might have possible biases. One of them being publication bias as the authors made no attempt to identify unpublished RCTs. Although we did perform a comprehensive search, we acknowledge it is possible to miss a single or few RCT, especially non-English RCT.

## Conclusion

Manual therapy has an efficacy in the management of CTTH that equals prophylactic medication with tricyclic antidepressant. At present no manual therapy studies exist for chronic migraine or chronic cluster headache. Future manual therapy RCTs on primary chronic headache should adhere to the recommendation of the International Headache Society, i.e. primary end point is headache frequency and secondary end-points are duration and intensity. Future manual therapy studies on CM with and without medication overuse is also warranted, since such studies do not exist today.

## Competing interests

The authors declare that they have no competing interests.

## Authors’ contributions

AC prepared the initial draft and performed the methodological assessment of the included studies. MBR had the original idea of the study, planned the overall design and revised the drafted manuscript. Both authors have read and approved the final manuscript.

## Authors’ information

Aleksander Chaibi is a BPT, MChiro, PhD student and Michael Bjørn Russell is a professor, MD, PhD, DrMedSci.
